# Laser-induced 2D/0D graphene-nanoceria freestanding paper-based films for on-site hydrogen peroxide monitoring in no-touch disinfection treatments

**DOI:** 10.1007/s00604-024-06427-9

**Published:** 2024-06-01

**Authors:** José M. Gordón Pidal, Selene Fiori, Annalisa Scroccarello, Flavio Della Pelle, Francesca Maggio, Annalisa Serio, Giovanni Ferraro, Alberto Escarpa, Dario Compagnone

**Affiliations:** 1 Present Address: Department of Analytical Chemistry, Physical Chemistry and Chemical Engineering, University of Alcalá, Alcalá de Henares, Madrid, 28871 Spain; 2 Department of Bioscience and Technology for Food, Agriculture and Environment, University of Teramo, Campus “Aurelio Saliceti” Via R. Balzarini 1, Teramo, 64100 Italy; 3 Department of Chemistry “Ugo Schiff” and CSGI, University of Florence, Via Della Lastruccia 3, Sesto Fiorentino, Florence, I-50019 Italy

**Keywords:** Laser-induced nanostructure, CO_2_-laser, Electrocatalysis, Real-time disinfection monitoring, Paper-based sensor

## Abstract

A one-shot CO_2_ laser-based strategy to generate conductive reduced graphene oxide (rGO) decorated with nanoceria (nCe) is proposed. The 2D/0D rGO-nCe films, integrated as catalytic sensing layers in paper-based sensors, were employed for on-site monitoring of indoor fogging treatments against *Listeria monocytogenes* (Lm), a ubiquitous pathogenic bacterium. The rGO-nCe laser-assisted synthesis was optimized to preserve the rGO film morphological and electron-transfer features and simultaneously integrate catalytic nCe. The films were characterized by microscopical (SEM), spectroscopical (EDX, Raman, and FTIR), and electrochemical techniques. The most performing film was integrated into a nitrocellulose substrate, and the complete sensor was assembled via a combination of xurography and stencil printing. The rGO-nCe sensor's catalytic activity was proved toward the detection of H_2_O_2_, obtaining sensitive determination (LOD = 0.3 µM) and an extended linear range (0.5–1500 µM). Eventually, the rGO-nCe sensor was challenged for the real-time continuous monitoring of hydrogen peroxide aerosol during no-touch fogging treatment conducted following the EU’s recommendation for biocidal product use. Treatment effectiveness was proved toward three Lm strains characterized by different origins, i.e., type strain ATCC 7644, clinical strain 338, and food strain 641/6II. The sensor allows for discrimination and quantification treatments at different environmental biocidal amounts and fogging times, and correlates with the microbiological inhibition, promoting the proposed sensor as a useful tool to modulate and monitor no-touch treatments.

**Graphical Abstract:**

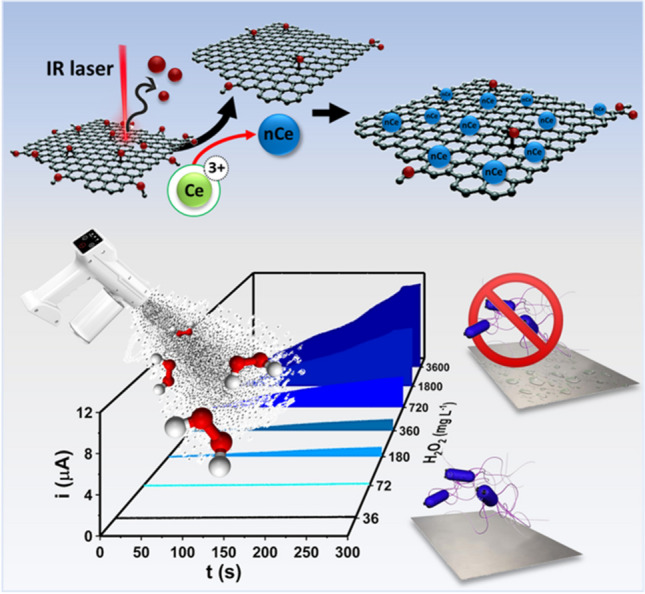

**Supplementary Information:**

The online version contains supplementary material available at 10.1007/s00604-024-06427-9.

## Introduction

In the last decade, sensor technology exhibited significant advances, in particular in diagnostics and food analysis/control, covering areas from safety monitoring to quality assessment; in this framework, nanotechnology has opened a new era giving rise to electrochemical sensors with improved/on-demand features [1]. Nonetheless, the nanomaterials (NMs) synthesis via bottom-up/top-down approaches and their integration into sensors still represent a critical issue. Indeed, NM synthesis is mainly based on multistep cumbersome procedures that need complex purification and dimension/shape selection steps; moreover, the post-synthesis manipulation to integrate NMs in sensors is often difficult and can hinder the full exploitation of their properties.

Benchtop laser-based technologies have been increasingly used for NMs patterning, structuring, and property-boosting ability toward carbonaceous and metal substrates/materials; they take advantage of the photothermal energy released by the laser IR emission which, in the case of photosensitive substrates, leads to instantaneous morpho-chemical changes [2, 3]. IR-laser plotters have been employed to produce graphene-like and graphitized surfaces/structures, directly treated with laser carbon-containing substrates [4]. Properly laser-produced NMs possess high nano structuration, large surface area, electrical conductivity, thermal stability, and chemical resistance, becoming captivating materials for sensor development; indeed, laser-based technologies can allow the on-demand and mask-free patterning of graphitized sensing surfaces, enabling the easy fabrication of sensors and devices [4]. Among others, laser-based strategies have been used to produce laser-induced graphene (LIG) and reduced graphene oxide (rGO) sensing films. In this case, the carbon source consists of non-conductive polyimide or graphene-oxide substrates, that via controlled laser irradiation are converted into conductive films, with chemistry and nanostructure consistent with the carbon source and laser parameters employed.

In addition, the laser proved the ability to drive nanodecoration and heterostructure formation; the latter approach allows for overcoming cumbersome and multistep approaches commonly employed [5], ensuring the formation of conductive nanocomposites with functional electrocatalytic features [6]. For instance, You et al. have co-synthesized LIG and noble metal nanoparticles (i.e., gold, silver, and platinum NPs) to construct an immuno-impedimetric sensor for *E. coli* O157:H7 [7]. Rodrigues et al. achieved the simultaneous formation of LIG and zinc oxide nanoparticles (NPs) via laser scribing [8], while the direct laser-induced formation of rGO integrating noble metal NPs (i.e., Au, Ag, and Pt NPs) has been reported by Scroccarello et al. [6]. Among NMs for sensing, there is a growing interest in metallic NPs with enzyme-mimicking activity, also named nanozymes [9]; despite PtNPs still largely employed for their well-known catalytic features, alternative nanoparticles of non-noble metals have started to be used thanks to their low cost despite their synthesis and integration in sensors result cumbersome.

In this framework, cerium NPs, also known as nanoceria (nCe), are increasingly studied; these NPs exhibit catalytic activity due to the coexistence of two switchable valence states, i.e., Ce^3+^ and Ce^4+^ [10]. Interestingly, the nCe catalytic ability toward redox processes varies according to the dimensions and aggregation state [11]. The catalytic properties of nCe have been exploited for the electrochemical detection of different analytes such as dopamine [12], hydrazine [13], uric acid [14], L-cysteine [15], NO_2_ [16], and H_2_O_2_ [17]; in these works, nCe or composites containing nCe were synthesized and later integrated into commercial electrodes through dedicated steps. Indeed, nCe is conventionally synthesized via hydrothermal methods, coprecipitation, sol-gel process, pyrolysis, laser ablation, or sonochemical methods, and then integrated into electrodes. These classical approaches show some drawbacks concerning the reaction time, high reaction temperatures, and solvent needs, as well as the use of external reducing and stabilizing agents which usually affect the nCe surface quality and features [9]. To the best of our knowledge, transferable freestanding films composed of conductive nanomaterials integrating nCe on board paper-based electrochemical sensors have been not yet reported.

Scientific evidence has proved that contamination of environmental surfaces in the food industry and nosocomial environments plays an important role in the direct transmission of pathogens and food contamination [18, 19]. In this framework, no-touch environmental disinfection technologies demonstrated their overall potential during the SARS-CoV-2 pandemic era, becoming the most affordable and rapid approach to ensure hygiene maintenance in closed environments. No-touch disinfection technologies consist of strategies where pathogen control is ensured without the need to fiscally touch “surfaces” to be sanitized. In particular, indoor biocidal fogging treatments can maximize the disinfectants’ effect thanks to the aerosol surface-to-volume ratio and ability to reach all exposed surfaces; this allows to overcome the limits of manual treatments (i.e., times, manpower, risk of spreading of pathogenic microorganisms) [19]. In this framework, EU recommendations regarding microbiological containment in food factories include no-touch treatments [20]. Among pathogenic bacteria, *Listeria monocytogenes* (Lm) is ubiquitous and able to contaminate biotic and abiotic surfaces [21], since possesses marked adaptive mechanisms and a wide strains-dependent resistance; only in 2022 Lm caused 2738 cases of illness and 286 deaths in humans [22]. Among others hydrogen peroxide room disinfection systems via handheld devices have become popular, in this frame, hydrogen peroxide fogging treatments have proved to be effective for Lm: interestingly, in this case, the Lm resistance is strongly strain-dependent [23], and for this reason, is recommendable to test different strains. In brief, nowadays fogging treatments are routinely used for healthcare ambients and food-industrial microbial control; for these reasons, easy-to-use and cost-effective equipment to manage, set, and monitor no-touch treatments is strongly required.

In the current study, a CO_2_ laser plotter-based strategy to produce in a few seconds a 2D/0D rGO-nCe catalytic film is proposed for the first time. The rGO-nCe film synthesis was optimized characterizing the morphological and electrochemical features; the film exhibits an enzymatic-like activity toward hydrogen peroxide, because of the nCe, while the rGO film acts as a hosting conductive network. The heterostructured rGO-nCe film has been integrated into complete paper-based lab-made sensors, that were challenged for on-site amperometric monitoring of no-touch environmental fogging treatments conducted according to EU recommendation for “food area” disinfection. The treatment effectiveness was evaluated towards Lm type strain, and Lm strains isolated from nosocomial environment and food matrix (smoked salmon). Fogging treatments to generate different environmental biocidal environments were used to prove the paper sensor exploitability and quantify the treatment intensity.

## Materials and methods

### Chemicals and materials

Sodium phosphate monobasic monohydrate (NaH_2_PO_4_), sodium phosphate dibasic anhydrous (Na_2_HPO_4_), potassium dihydrogen phosphate (KH_2_PO_4_), potassium ferrocyanide ([Fe(CN)_6_]^4−^), potassium ferricyanide ([Fe(CN)_6_]^3−^), potassium chloride (KCl), sodium chloride (NaCl), hexaammineruthenium(III) chloride ([Ru(NH_3_)_6_]^3+^), hexamineruthenium(II) chloride ([Ru(NH_3_)_6_]^2+^), cerium(III) nitrate hexahydrate (Ce^3+^), 30% w/w hydrogen peroxide solution (H_2_O_2_), and glycerol were purchased from Sigma-Aldrich (St Louis, MO, USA). Tryptic Soy Broth (TSB), Tryptic Soy Agar (TSA), and yeast extract were purchased from Liofilchem (Roseto degli Abruzzi, Italy), Agar Listeria according to Ottaviani Agosti (ALOA) was purchased from Biolife Italiana (Milan, Italy).

Milli-Q water (18.2 MΩ) was used for solution preparation and experiments. A stock solution of 50 mM Ce^3+^ was prepared in water and stored in the fridge at + 4 °C. Phosphate buffer saline (PBS) pH 7.4 for microbiological experiments was prepared with 8.0 g L^−1^ NaCl, 0.2 g L^−1^ KCl, 1.4 Na_2_HPO_4_, and 0.2 g L^−1^ KH_2_PO_4_.

10 mg mL^−1^ graphene oxide (GO) dispersion (N002-PS-1.0) was purchased from Angstrom Material (Dayton, USA). Nitrocellulose coils (NTR) with 8Dm pores were purchased from advanced Microdevices (Ambala, India). Silver paste ink (Ag-ink, C2180423D2) and grey dielectric paste (D2070423P5) were purchased from Gwent group/Sun Chemical (Pontypool, U.K.). PVDF membrane (0.1 μm of pore size, 47 mm of diameter) was purchased from Millipore (Massachusetts, USA), adhesive vinyl stencil mask was purchased from TINYYO (Parson Drove, Wisbech, U.K.).

### Apparatus

A detailed description is reported in *section SM.2.2*.

### Paper sensors integrating laser-conceived rGO-nCe catalytic freestanding films manufacturing

The sensors were manufactured as sketched in Scheme S1 according to the procedure described by Silveri et al. [24] with some modifications. Conductive contacts and the reference electrode (RE) were printed on nitrocellulose strips (NTR) using a vinyl stencil molded by a cutter-plotter (blade depth: 3, blade power: 12, blade speed: 10); distances between contacts were designed to be compatible with commercial potentiostats. The adhesive stencil mask was adhered onto NTR and the Ag-ink was spread evenly through a squeegee; then, the stencil mask was removed, and the Ag-ink was cured at 100 °C for 10 min (Scheme S1A).

The working (WE) and the counter (CE) electrode were composed of the rGO-nCe film; the film was obtained by vacuum-filtering on a PVDF membrane 5 mL of 1 mg mL^−1^ GO dispersed in 10 mM Ce^3+^ aqueous solution, previously homogenized with an orbital shaker (1 min, 300 rpm). The GO-Ce^3+^ film was let dry (at room temperature) and then nanostructured with the laser in engraving mode (laser power/ LP = 2.1 W, laser speed/ LS = 1.5 m s^−1^) according to the WE and CE design. The electrode pairs (WE + CE) were cut by laser in cutting mode (LP = 1.8 W, LS = 4.5 × 10^−2^ m s^−1^); 11 WE (Ø= 3 mm) and CE of rGO-nCe were obtained from each membrane (Scheme S1B).

The rGO-nCe electrode pairs were then aligned with the electrical contacts and transferred onto NTR support (1 × 2.5 cm) by a hydraulic press (2.0 ± 0.2 tons, 3 min). Eventually, the sensors’ contacts were insulated with the dielectric paste and cured at 100 ºC for 15 min (Scheme S1C).

### Morphochemical and electrochemical characterization

A detailed description is reported in *section SM.2.4*.

### Bacterial strains and cultural conditions

A detailed description is reported in *section SM.2.5*.

### rGO-nCe sensor no-touch disinfection continuous monitoring. *L. monocytogenes* as a case study

rGO-nCe sensors were challenged for the real-time continuous monitoring of H_2_O_2_ aerosol during no-touch fogging treatment; the effectiveness of the treatment was proved towards different strains of *L. monocytogenes* (*section SM.2.5*).

Tests were carried out using as an environmental model a 1 m^3^ sealed fogging box placed in a 14 °C thermostat room; a portable nano-atomizer from Migaven was placed in the box and employed to fog the H_2_O_2_. The rGO-nCe sensor was placed in the box closely next to the stainless steel coupons containing Lm; for each strain, three Petri dishes containing coupons in triplicate equally positioned were employed. The sensor and the coupons were placed behind the atomizer nozzle to receive only the H_2_O_2_ fogged; Scheme S2 reports a sketch of the fogging box.

Before starting the fogging treatment, 40 µL of 0.1 M phosphate buffer pH 7.0 (PB) was absorbed on the sensor cellulose measuring space containing the electrochemical cell, and amperometry at + 0.4 V was run. The H_2_O_2_ fogging treatment was initiated 2 min after starting the amperometry to allow signal stabilization; before measurement, sensors were conditioned at + 0.8 V in PB for 1 min. The H_2_O_2_ solution was nebulized at 5.5 mL min^−1^, and various concentrations of H_2_O_2_ working solution and fogging times were tested to simulate different treatment intensities (“rGO-nCe paper sensor no-touch disinfection continuous monitoring” section). Once the treatment, the fogging box was left closed for 90 min according to the EU’s recommendation for biocidal product use [20].

The disinfection treatment effectiveness was evaluated according to Møretrø et al. [19] with some modifications. In brief, after the fogging treatment, the stainless steel coupons containing Lm were swabbed with a sterile cotton swab pre-moistened with saline solution (0.85% w/v NaCl) and then transferred in a tube with neutralizing solution according to Haines et al. [25]. The viable bacterial count was performed by plating onto TSA added with 6 g L^−1^ yeast extract and incubating at 37 °C for 24–48 h. The treatment efficacy was evaluated by comparing the colony-forming units (CFU) of treated Lm strains with the control, intended as the respective non-treated Lm strain incubated in the same condition.

## Results and discussion

Here, a straightforward strategy to synthesize in one-step 2D/0D rGO-nCe freestanding films is proposed. The rGO-nCe film was integrated into lab-made complete paper sensors that combine the catalytic features of nanoceria with the high electron transfer ability of the graphene nanonetwork, enabling enzyme-free H_2_O_2_ detection. rGO-nCe-based paper sensors were challenged for the on-site continuous monitoring of no-touch fogging treatment aimed at food-contact surface disinfection, using three different *L. monocytogenes* strains with different origins as a model.

### 2D/0D nanostructured freestanding film laser-assisted synthesis and characterization

To obtain 2D/0D catalytic transferable nanostructured films, the CO_2_ laser’s ability to induce GO conversion to conductive rGO and to drive, at the same time, the synthesis of nanoceria was initially explored. Since the synthesis has been never attempted using a CO_2_-laser, the amount of nanoceria precursor (Ce^3+^) was studied, while the GO amount and laser parameters were set according to our previous studies [2, 6]. GO and different amounts of Ce^3+^ were co-filtered and the obtained films were treated with the laser. The resulting 2D/0D-hybrid nanostructures were then transferred onto nitrocellulose containing contacts and reference electrodes, and assembled in complete sensors; the detailed rGO-nCe synthesis and the nanofilm integration in paper sensors are reported in the “Paper sensors integrating laser-conceived rGO-nCe catalytic freestanding films manufacturing” section and graphically summarized in Scheme S1.

**Fig. 1:**
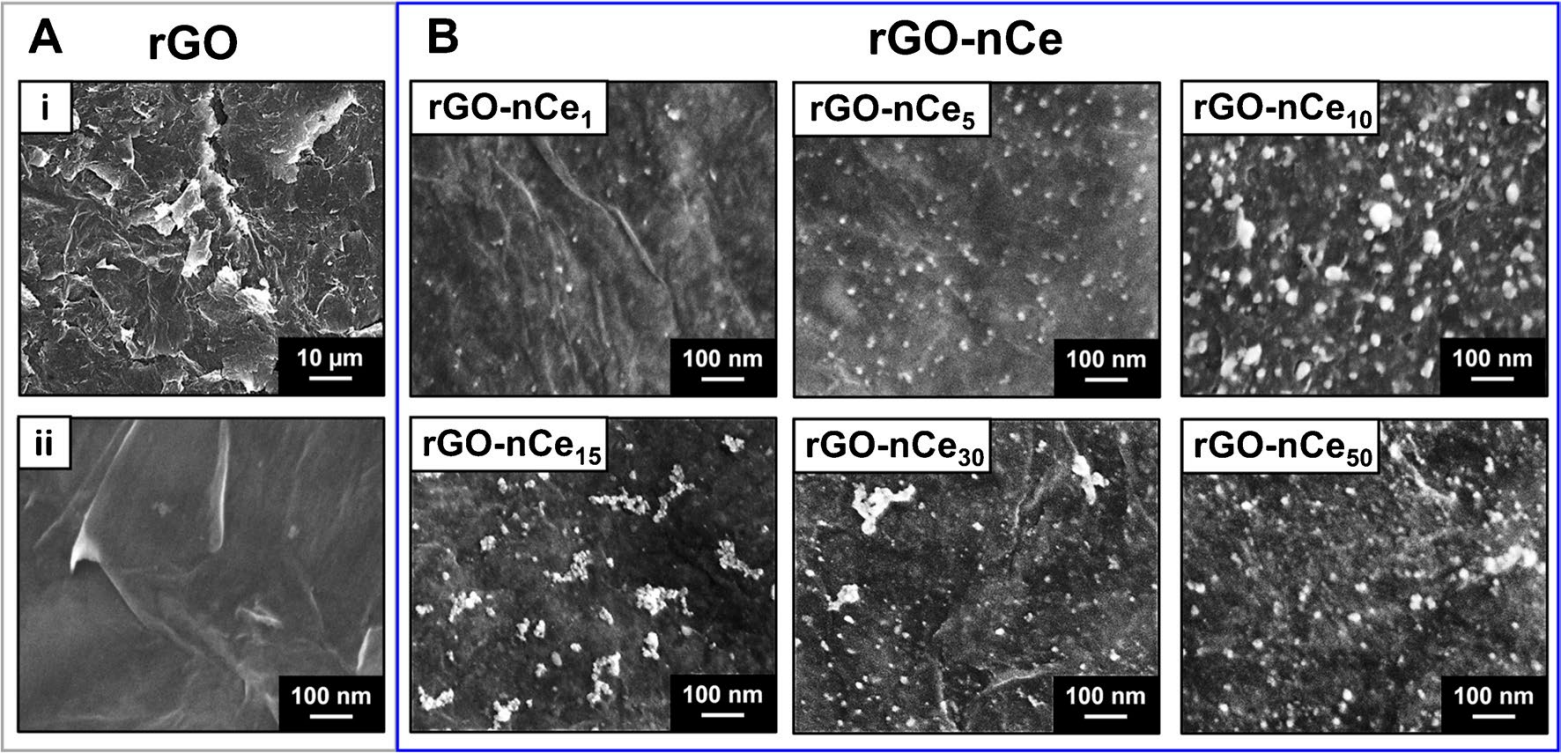
SEM micrographs of **A** rGO film acquired at Mag 500x (**i**) and 50 Kx (**ii**). **B** Micrographs of rGO-nCe films obtained using Ce^3+^ concentrations of 1, 5, 10, 15, 30, and 50 mM; micrographs acquired using an InLens detector acquired at Mag 50 Kx

The films of rGO-only and rGO-nCe obtained using 1, 5, 10, 15, 30, and 50 mM of Ce^3+^ were morphologically characterized through scanning electron micrographs (Fig. 1**)**.

Figure 1A-i demonstrates that the laser-induced rGO film appears wrinkled, with a multilamellar nanostructure associated with highly exfoliated flakes characterized by sharply/edgy profiles with nanometric thickness. In the nano-magnification of Fig. 1A**-ii**, it is possible to appreciate how the single rGO flakes, as expected, appear uniform, continuous, and smooth. On the contrary, the GO film, before the laser treatment, appears flat and in-plane due to the stacking of graphene oxide sheets (data not shown). Macroscopic evidence of the laser-induced GO conversion to rGO can be easily appreciated in pictures reported in Scheme S1B, while the morphochemical changes are discussed later in this section.

On the other hand, the micrographs of rGO-nCe nanofilms reported in Fig. 1B prove the production of the ceria nanoparticles (nCe) that are anchored onto the rGO that acts as a hosting substrate. In all the cases, nCe possesses a spheroidal structure comprised in the nanodomain, and results homogeneously distributed on the rGO surface. Noteworthy, the morphology of the nCe in the films is dependent on the precursor amount: the nCe size increases up to 10 mM, resulting of 8.1 ± 3.3 nm, 13.5 ± 3.4 nm, and 29.9 ± 10.6 nm for rGO-nCe_1_, rGO-nCe_5_, and rGO-nCe_10_, respectively; higher precursor amounts (Ce^3+^≥15 mM) lead to nCe clusterization and the formation of polydisperse NPs. The nitrocellulose support used as a base for the nanofilms transfer allows the preservation of the rGO and rGO-nCe nanostructure avoiding extensive flake restacking. The nitrocellulose porosity can accommodate the nanostructured film.

**Fig. 2:**
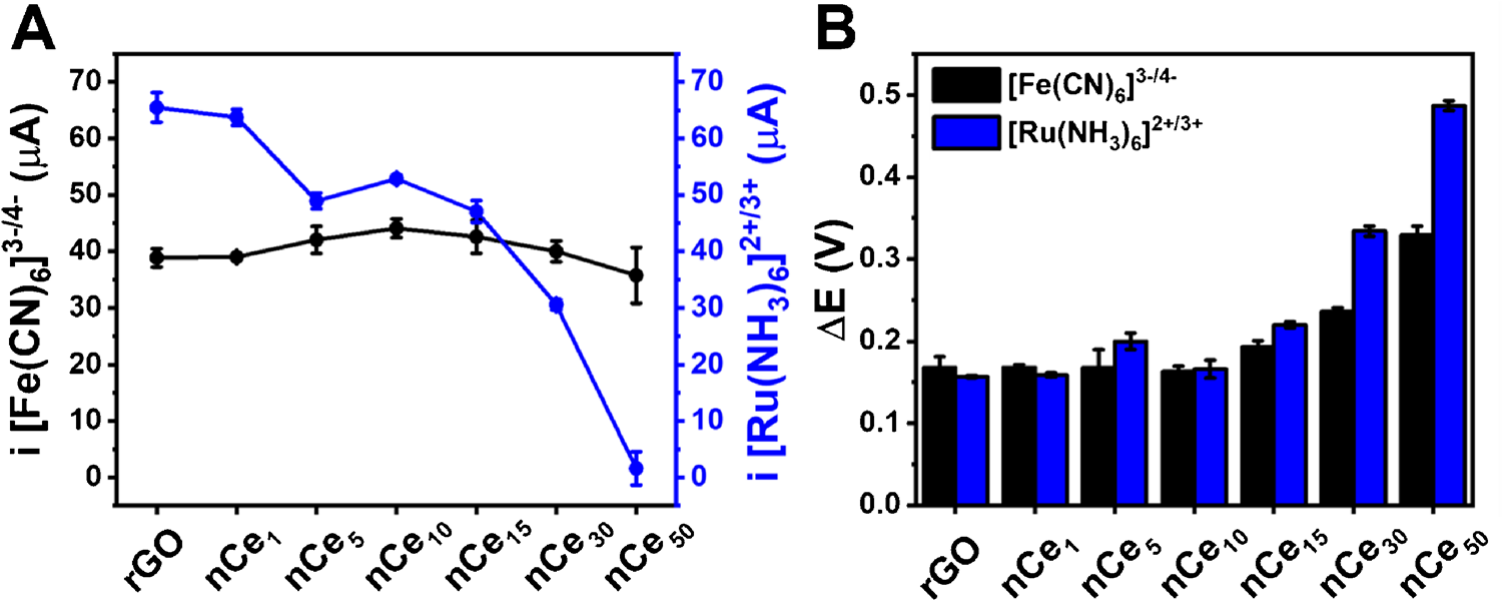
**A** Current intensity of the anodic peak and **B** peak-to-peak separation (ΔE) collected from cyclic voltammetry carried out in 5 mM [Fe(CN)_6_]^3−/4−^ in 0.1 M KCl (**black**) and 5 mM [Ru(NH_3_)_6_]^2+/3+^ in 0.1 M KCl (**blue**) at 25 mV s^−1^, using rGO and rGO-nCe sensors obtained with different amounts of Ce^3+^ precursor reported as subscript (mM). GO sensor has been not taken into account since it was not conductive

Then, rGO and rGO-nCe-based sensors were investigated using an inner ([Fe(CN)_6_]^3−/4−^) and an outer ([Ru(NH_3_)_6_]^2+/3+^) redox probe. Figure 2 reports the peak intensity (**A**) and the peak-to-peak potential separation (ΔE) (**B**) achieved from cyclic voltammetry (CV); Figure S1 displays the voltammograms.

The two redox probes were selected since [Fe(CN)_6_]^3−/4−^ (inner sphere model) is sensitive to the electroactive sensor surface, and allows the investigation of electron transfer mechanisms that take place on the electrode surface; whereas [Ru(NH_3_)_6_]^2+/3+^ (outer sphere model) is less sensitive to the electrode surface features since the electron transfer occurs through the interlayer solution among the probe and the electrode surface [26].

It is possible to appreciate that [Fe(CN)_6_]^3−/4−^ presents a constant peak intensity independent of the nCe concentration (Fig. 2A), while ΔE shows a significant increase for nCe precursors ≥ 15 mM (Fig. 2B). On the contrary, the [Ru(NH_3_)_6_]^2+/3+^ peak intensity decreases concerning the Ce^3+^ amount used for the synthesis of the film, with a sharp decrease starting at rGO-nCe_15_; also in this case, ΔE gets larger for nCe precursors concentration ≥ 15 mM.

nCe belongs to the class of metal oxide NPs and, concerning noble metal NPs [10] hinders the electron transfer capacity of carbonaceous material because the oxygen surface groups act as “non-conductive pits” [27]. In this case, the [Ru(NH_3_)_6_]^2+/3+^ ability to react on the surface of the sensor is negatively influenced by the non-conductivity of nCe, which affects the ability to exchange charge across the solvent layer; instead, the [Fe(CN)_6_]^3−/4−^ electron transfer directly occurs by adsorption of the species onto the sensing surface; thus, for a low amount of nCe, the high “conductive” surface of rGO predominates, while in the presence of the high amount of nCe and its clusters (from nCe_15_) the electron transfer capacity is affected [26].

The observed electrochemical behavior was confirmed by extrapolating the electroactive surface area (ECSA) according to Randles–Sevick’s equation [28] and calculating the heterogeneous electron transfer constants (k^0^) according to the Nicholson method [29], using [Fe(CN)_6_]^3−/4−^ as a probe. No significant ECSA differences were observed among rGO-only and the rGO-nCe sensors (RSD = 8%, *n* = 7) with an average ECSA of 9.74 ± 0.76 mm^2^. On the other hand, k^0^ values similar to rGO have been recorded up to the sensor rGO-nCe_10_ (average K^0^ = 1.9 × 10^−3^; RSD = 3% for different sensors), while for higher amounts of Ce^3+^, an electron transfer decrease was observed (rGO-nCe_30_ K^0^ = 1.2 × 10^−3^; rGO-nCe_50_ K^0^ = 0.6 × 10^−3^).

From the obtained data, rGO-nCe_10_ results in the best compromise between high loading of nCe and preserved rGO electrochemical features; moreover, this sensing surface microscopically shows the highest nCE density without the presence of clusters. As will be shown in the next section, this sensor will result also the most catalytic toward hydrogen peroxide.

**Fig. 3:**
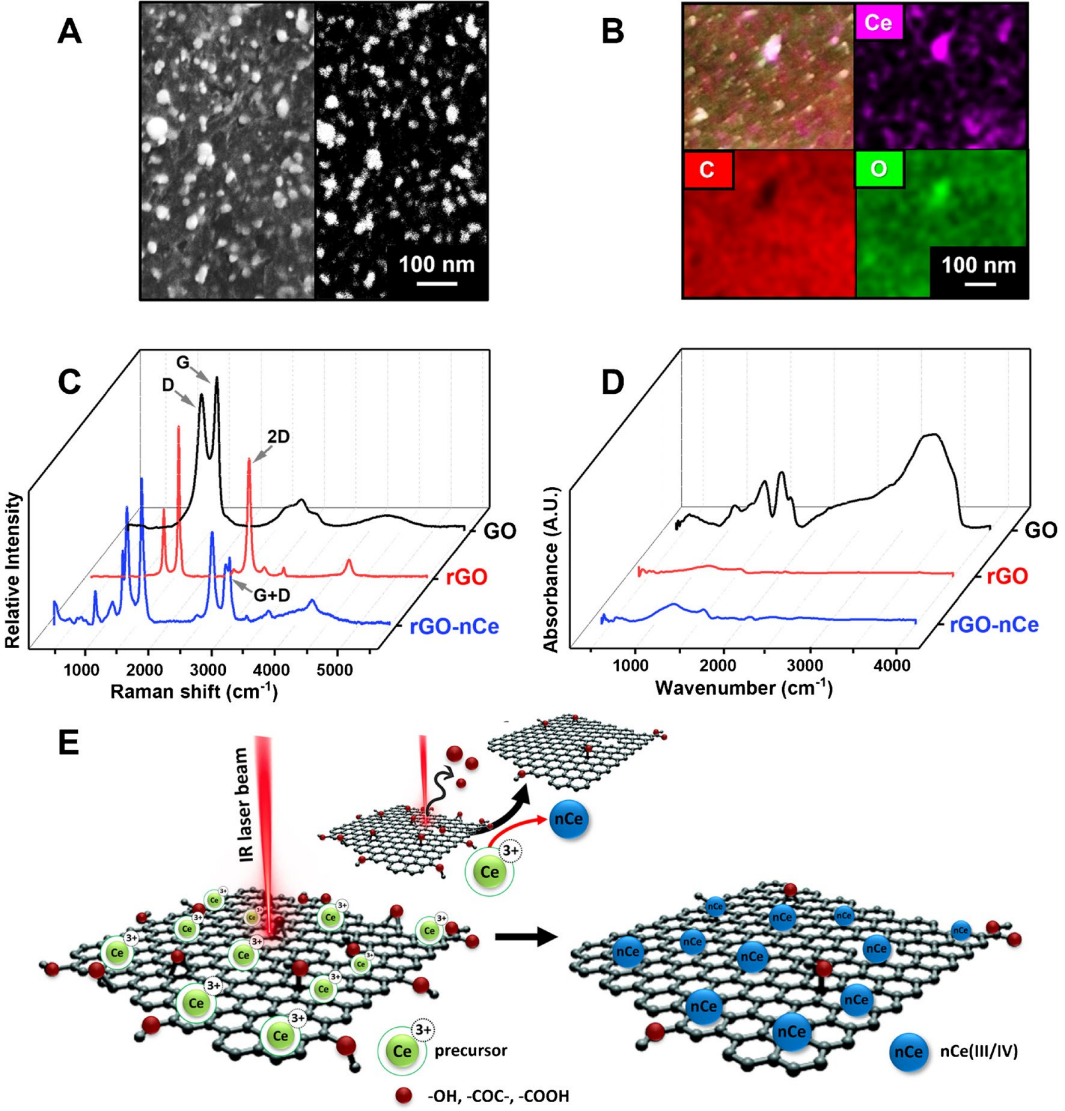
**A** SEM micrographs of the rGO-nCe_10_ film acquired at Mag 50 Kx using an In-Lens (right) and EsB (left) detector. **B** Ce, C, and O elemental mapping by EDX for the rGO-nCe_10_ film. **C** Raman spectra of GO (black line), rGO (red line), and rGO-nCe_10_ (blue line) films. **D** FTIR spectra were acquired in the transmission mode of GO (black line), rGO (red line), and rGO-nCe_10_ (blue line) films. **E** Graphical sketch of the rGO-nCe film CO_2_ laser-induced formation

To shed light on the formation mechanism of the 2D/0D nanoarchitecture, characterizations were performed via microscopy (SEM) and spectroscopical (Raman, EDX, FT-IR) analysis on the rGO-nCe_10_ system. Figure 3A reports the same micrograph acquired using an In-Lens detector (left) and EsB detector (right); Fig. 3A**-left** demonstrates how nCe is widely distributed and well anchored to the rGO flakes, while, in the SEM micrograph acquired by the EsB detector (Fig. 3A**-right**), the nCe results “packed” along the rGO film thickness and the particles result formed by heavy elements.

The nCe presence was confirmed by EDX elemental analysis, where the nCe oxide nature was confirmed by elemental mapping reported in Fig. 3B, which highlights how oxygen is particularly present in correspondence with nCe. This is further confirmed in Figure S2 where EDX spectra evidence an increase in the oxygen content in the rGO-nCe concerning the rGO.

To gain more information about the rGO-nCe film chemistry and structure, Raman spectra of GO, rGO, and rGO-nCe films were also recorded. Figure 3C, showing the Raman spectra of rGO and rGO-nCe, the laser-induced GO reduction is confirmed by the relative D band decrease (1350.9 ± 1.9 cm^−1^) and 2D band appearance (2689.6 ± 1.0 cm^−1^), compared to the rGO only; these changes can be attributed to the sp^3^ carbon/oxygen-containing groups removal with the following restoration of sp^2^ domains and the exfoliation/bidimensional structural rearrangement [2, 6]. In particular, the GO possesses the highest I_D_/I_G_ ratio (0.88 ± 0.05), while the lowest value was observed for the rGO (I_D_/I_G_ = 0.45 ± 0.03), confirming the rise of the sp^2^ carbon domain. In the case of rGO-nCe, the I_D_/I_G_ ratio is 0.75 ± 0.06: this could be ascribed to the chemical defects and modification induced by the formation of nCe and to the reduction of both GO and Ce^3+^ induced by the photothermal energy of the IR laser beam. In addition, the spectrum of rGO-nCe is significantly more complex than the rGO-only, confirming the presence of other contributions beyond the graphenic carbon. In particular, the appearance of a prominent G + D band, centered around 2903.47 ± 5.62 cm^−1^, suggests the introduction of defects on the rGO profile. The nCe presence on the film is further confirmed by the characteristic bands observed in the range 250–700 cm^−1^ (Figure S3A) induced by the Ce stretching centered at around 409.3, 446.7, 571.1, and 629.9 nm commonly reported for polycrystalline nCe structures [30].

Figure 3D depicts the FTIR spectra obtained for GO, rGO, and rGO-nCe systems. GO spectrum appears significantly different compared to rGO and rGO-nCe and is characterized by graphitic skeleton vibrational stretching (centered at 530 and 1592 cm^−1^ for C-H and C = C, respectively), C-O-C epoxydic stretching (1050 cm^−1^) and C = O carbonyl (1700 cm^−1^) groups; the GO chemistry is confirmed by the characteristic prominent stretching vibrations observed around 3340 cm^−1^ due to OH hydroxylic group [31, 32]. After laser treatment, the rGO signature appears devoid of oxygen groups, highlighting only vibrations attributable to graphenic structure [33]; the same trend is observed for rGO-nCe, where C = C and C-H stretching are observed at around 1560 and 2400 cm^−1^, respectively (Figure S3B). In the latter case, the different GO reduction is pointed out by binding vibrations of epoxy (1200 cm^−1^) and carbonyl (1735 cm^−1^), probably given by the “non-complete” GO reduction caused by the nCe formation/presence. More interestingly, a characteristic spectrum zone ranging from 400 to 700 cm^−1^ shows a vibrational stretching profile that can be attributed to Ce-O bonds, suggesting the presence of cerium oxide [32].

Summing up, the nanostructured 2D/0D film formation occurs via a CO_2_ laser-driven co-reduction of the GO and nCe precursor (Ce^3+^) sketched in Fig. 3E. According to previous works of our group [6, 34], we can state that the IR laser beam (λ = 10.6 µM) triggers the photothermal reduction of the oxygen-containing groups (-COOH, -COC-, -COH, etc.) along the GO film, inducing the sp^2^ domain recovery and rGO formation. The removal of oxygen functionalities gives rise to graphene sheets destaking and cracking, which results in wrinkled flake-structure formation (see Fig. 1A). Simultaneously, the Ce^3+^ adsorbed onto the GO is converted into ceria-nanoparticles (nCe), thanks to the excess of photothermal energy provided by the laser and the electron-rich surface of GO; the latter acts also as nucleation center favoring the nCe production and anchoring.

### rGO-nCe film electrocatalytic features and paper sensor performance

It is well known that nCe may have catalytic properties, dependent on the synthesis procedure, dimensions, and the hosting conductive substrate [35]. The catalytic features brought by the presence of nCe were investigated in the presence of hydrogen peroxide via cyclic voltammetry (CV) and amperometry.

**Fig. 4:**
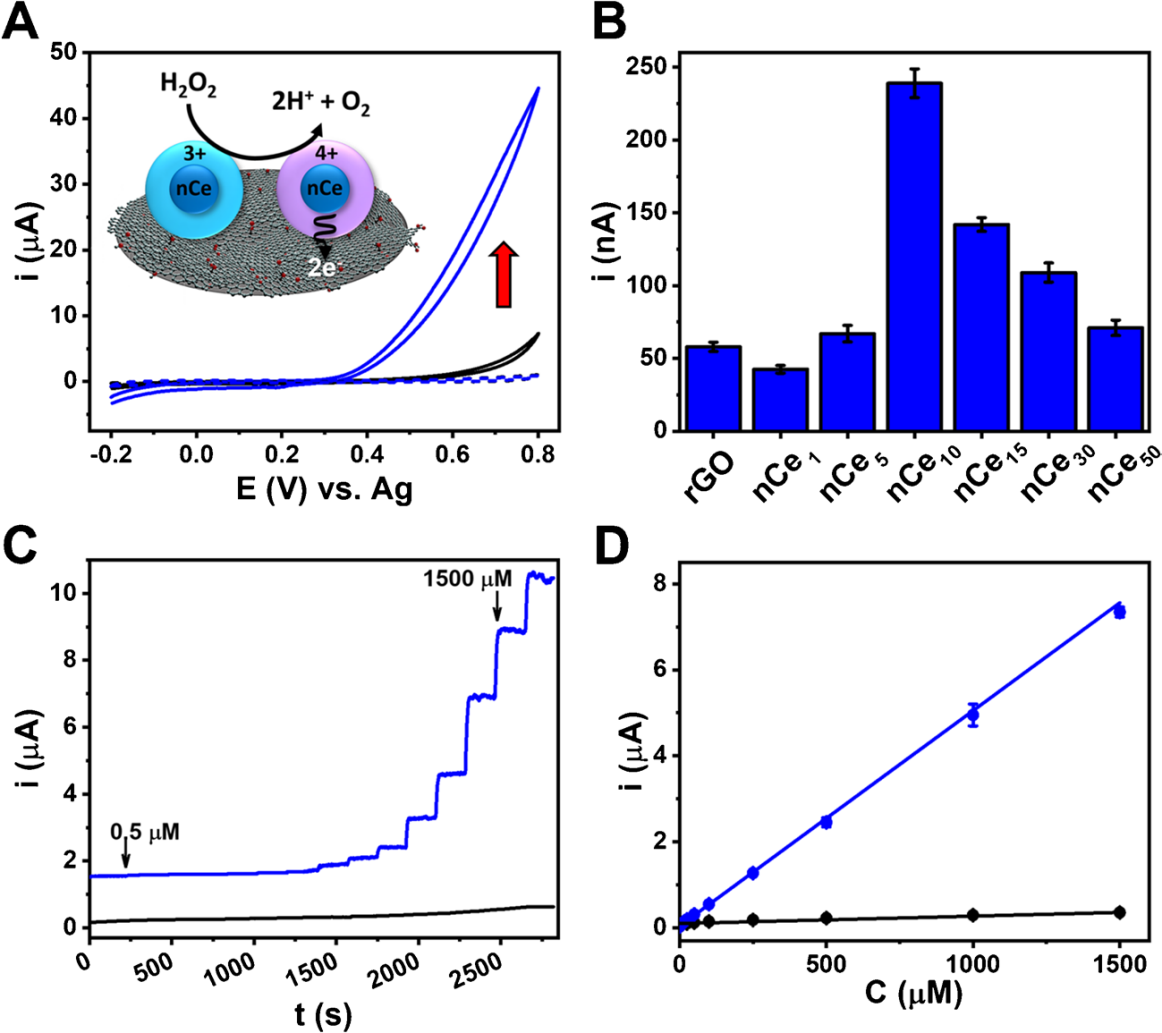
**A** Cyclic voltammograms for rGO (black line) and rGO-nCe_10_ (blue line) sensors performed in 10 mM H_2_O_2_ in PB, and cyclic voltammogram of rGO-nCe sensor acquired in PB only (dashed blue line); scan rate 25 mV s^−1^. The inset graphically resumes the catalytic mechanism of nCe toward hydrogen peroxide. **B** Amperometric currents obtained in presence of 50 µM H_2_O_2_ for rGO and rGO-nCe_1−15_ sensors E = + 0.4 V vs. Ag. **C** Amperometry measurements carried out at +0.4 V increasing the amount of H_2_O_2_; the arrow indicates the linear range (0.5 to 1500 μM) for the rGO-nCe_10_. **D** Linearly fitted data for the rGO (black line; L.R. 25–1500 μM, linear fit eq: y = 0.173 [± 5.9 × 10^−3^] x + 97.222 [± 1.181]; R^2^ = 0.9943) and rGO-nCe_10_ (blue line; L.R. 0.5–1500 μM, linear fit eq: y = 5.014 [± 0.088] x + 38.760 [± 3.315], R^2^ = 0.9985) sensor. The relative measurement performed in the buffer was subtracted from each signal

Figure S4  shows the CV profile obtained for the full set of sensors: for all the sensors containing nCe, a significant rise in anodic current was highlighted, with the onset potential around + 0.25 V; this behavior was not observed for rGO. This catalytic behavior can be fully appreciated in Fig. 4A, where the voltammogram for rGO-nCe_10_ obtained in the presence of H_2_O_2_ is reported.

The observed prominent anodic catalytic current (blue solid line) can be attributed to the rGO-nCe_10_ that acts as a catalyst for the oxidation of H_2_O_2_ at applied voltage. The same behavior is not observed for the rGO without ceria (black solid line). Figure 4A reports the suggested mechanism of catalysis, where the H_2_O_2_ is oxidized to O_2_, and the electrochemical current increase can be attributed to the electrons release (2e^−^) caught by the graphenic sensing surface [11, 17]. This enzymatic-like activity is related to the nCe surface chemistry, where the reaction occurs thanks to the Ce^4+^ and Ce^3+^ cations and oxygen vacancies. According to Kosto et al. [11], the catalytic process can be resumed as follows: (i) H_2_O_2_ adsorption on Ce^3+^ via oxygen atoms, (ii) oxidation of Ce^3+^ to Ce^4+^ due to oxygen adsorption, (iii) then the Ce^4+^ with adsorbed oxygen specie is oxidized to O_2_ and the Ce^3+^ cations are produced again; the relative electrons give rise to the catalytic current recorded. Noteworthy, this catalytic activity is due only to Ce in the nanoceria form, in fact the cerium oxide surface chemistry is the key of this reactivity [36, 37].

Amperometric measurements of H_2_O_2_ were then attempted. Figure 4B shows the current intensities collected from the amperometric measurements of 50 µM H_2_O_2_ performed at + 0.4 V at rGO and rGO-nCe. rGO-nCe_10_ returned the larger current intensity (239.0 ± 7.2 nA) and, therefore, it was selected for further experiments; as reported in the “2D/0D nanostructured freestanding film laser-assisted synthesis and characterization” section, this can be attributed to the best compromise between nCe loading/density and preservation of the electron transfer capacity of the rGO network. To select the working potential, a hydrodynamic study in the potential range 0.0 V – +1.0 V was performed; the obtained current values are reported in Figure S5A. A linear increase in current response was observed at increasing potential, thus + 0.4 V was selected as a compromise between overpotential applied and signal intensity; for all the potential studied reproducible results were obtained (RSD ≤ 6%, *n* = 3).

Using the rGO-nCe_10_ and rGO as control, a dose-response analysis via amperometry analyzing H_2_O_2_ between 0.5 and 2000 µM was carried out. The obtained amperometric signals for rGO and rGO-nCe_10_ are reported in Fig. 4C; the superior performance of the nCe-containing sensor is evident. To better appreciate the sensor differences, Fig. 4D reports the dose-response linear fitting. The rGO-nCe_10_ returns a linear response between 0.5 and 1500 µM, with linear regression y/nA = 5.0143 [± 0.0878] x/µM + 38.7600 [± 3.3145]/nA (R^2^ = 0.9990); measurements were performed in triplicate and satisfactory inter-electrode reproducibility was observed (RSD ≤ 7%, *n* = 3). A limit of detection (LOD) of 0.3 µM was obtained for the rGO-nCe_10_, where LOD = [(3 × σ)/m], σ is the standard deviation of the y-intercept, and m is the linear regression slope. Furthermore, by analyzing rGO-nCe_10_ sensors produced by different batches (i.e., different films from which the catalytic film was produced), a reproducible sensitivity was also achieved (RSD ≤ 14%, *n* = 3).

Eventually, the stability of the rGO-nCe_10_ sensors was studied, performing calibration curves weekly, and comparing the linear regression slope (Figure S5B). The sensor performance slightly decreased during the first two weeks (RSD = 7%, *n* = 3) and then remained stable for about two months in which a more than satisfactory stability was obtained with RSD = 3% (*n* = 10). This result is particularly appreciable since often nCe is affected by physicochemical state variations, that may affect the performance of the catalyst [35].

An overview of the state of the art concerning nCe used in electrochemical sensors is reported in Table S1. Qiu et al. [38] proposed a sensor made of myoglobin capped nCe further coated with multiwalled carbon nanotubes, for the determination of H_2_O_2_ reporting a LOD of 0.2 µM; for the same analyte Zhang et al. [39] proposed a sensor made of hemoglobin immobilized on a CeO_2_/carbon nanotubes nanocomposite reporting a LOD of 0.7 µM. nCe was used as a modifier mainly for commercial electrodes, but not to produce transferable freestanding films composed of conductive nanomaterials, and nCe paper-based electrochemical sensors are not reported. Regardless of the sensor and material used, higher or comparable LODs are reported concerning the proposed sensor, moreover only in a few cases cerium-containing sensors have been used for real applications. Overall, the sensor preparation still presents a limit, commercial electrodes are mainly needed as transducers, the use of biological elements or other catalytic material is often required, and dedicated cumbersome synthesis and immobilization procedures are necessary for the fabrication of sensors containing nCe.

### rGO-nCe paper sensor no-touch disinfection continuous monitoring

Defining adequate disinfection procedures is a biosafety requirement, which also has economic implications, concerning the waste of biocidal agents and the potential damage that an oxidant may have on tools, machinery, and structures. In this framework, the exploitability of the rGO-nCe_10_ sensor was challenged to monitor disinfection treatment by “no-touch” technology; tests were conducted according to EU recommendatons in the framework of microbiological containment in food factories [20].

The tests carried out were aimed at evaluating the usability of the sensor to monitor fogging treatments carried out in different conditions, assessing the reproducibility of the monitoring data obtained, and correlating them to the microbiological effect. Since no analytical quantification of unknown samples has been performed, it was not possible to calculate the accuracy; since study in complex matrices was not required, and the analyte was present in water, no studies of interferents were performed. However, fogging treatments in absence of H_2_O_2_ have been attempted for the different tested times, without no recordable signals.

In particular, the sensors were challenged to monitor automated indoor environmental disinfections via fogging, using hydrogen peroxide as a broad-spectrum biocidal agent [19]; the effectiveness of the treatment was evaluated by studying the growth inhibition of three Listeria monocytogenes (Lm) strains ubiquitous and characterized by different resistance to disinfectants, i.e., Lm ATCC 7644 (type strain), Lm 338 (clinical strain), and Lm 641/6II (food-isolate strain); the complete description of the bacteria types and their ‘handling’ is detailed in section SM.2.5.

Fogging tests were carried out by varying the concentration of biocidal agent and nebulization times; the measurement setup employed is described in the “rGO-nCe sensor no-touch disinfection continuous monitoring. L. monocytogenes as a case study” section. In brief, the fogging box was thermostated to simulate a food-factory environment, and the rGO-nCe_10_ sensor and the stainless steel coupons containing the different Lm strains inocula were placed closely; once the measurement box, the nano-atomizer was activated. The electrochemical measurement was performed during the fogging via amperometry, and the area under the current/time curve, the electric charge (Q; µA s) [40], was employed to interpret the efficiency of treatment. At the end of each treatment, the Lm strains were recovered from the coupons, and the inhibition degree evaluation was carried out as reported in the “rGO-nCe sensor no-touch disinfection continuous monitoring. L. monocytogenes as a case study” section.

**Fig. 5:**
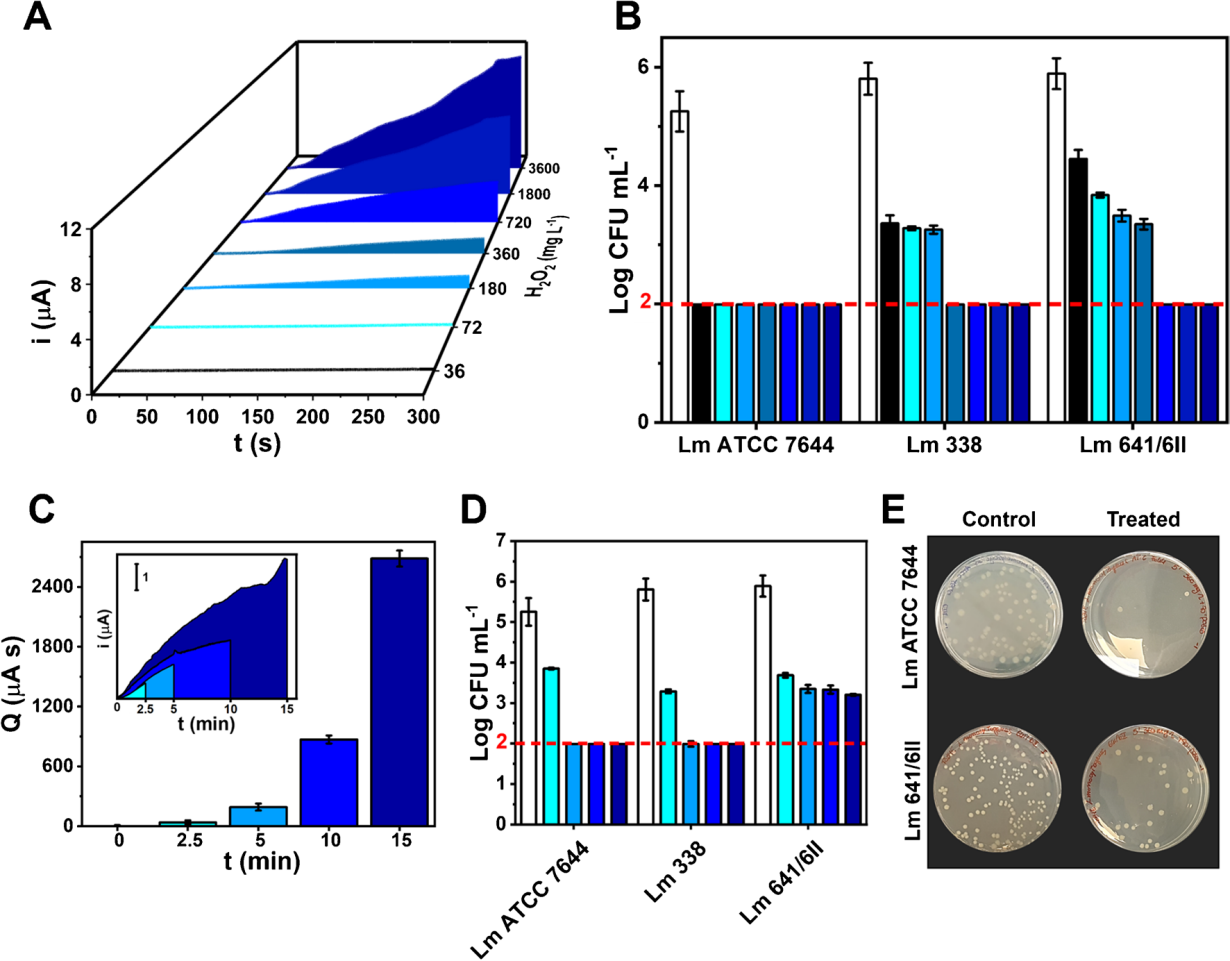
**A** Amperometric curves obtained using the rGO-nCe_10_ sensor (+0.4 V vs. Ag) during 5 min fogging treatments, performed to ensure increasing environmental concentrations of the nebulized H_2_O_2_. The different areas subtended by the current/time curve, used to calculate the electric charge (Q) are highlighted in different colors. **B** Inhibition degree of the different Lm strains caused by the disinfection treatment performed with increasing concentrations of H_2_O_2_. **C** Electric charge values (Q) extrapolated from fogging treatments performed keeping constant the H_2_O_2_ environmental concentration (i.e., 360 mg mL^−1^) and varying the nebulization time; the inset reports the relative amperometric curves, from which *Q* values were extrapolated. **D** Inhibition degree of the different Lm strains caused by the disinfection treatment performed varying the nebulization time. **E** Pictures of growth inhibition of the Lm ATCC 7644 and Lm 641/6II strains caused by the fogging treatments performed according to EU recommendations (i.e., 5 min nebulization to reach 360 mg L^−1^ H_2_O_2_) concerning the control experiments (untreated *Lm* strains)

Figure 5 reports the amperometric signal recorded with the rGO-nCe_10_ sensor for fogging treatment performed to ensure H_2_O_2_ environmental concentration of 36, 72, 180, 360, 720, 1800, and 3600 mg L^−1^; to achieve this, H_2_O_2_ different working solutions were employed, keeping constant the nano-atomizer flow (5.5 mL min^−1^) and the nebulization time (5 min)

Figure 5A demonstrates how the sensors can distinguish among the different treatments driven by the hydrogen peroxide increased concentrations, giving increasing amperometric currents along treatment time. Figure S6 reports the electric charge (Q) extrapolated from the area subtended by the amperometric curves (shown with different colors in Fig. 5A). Interestingly, the Q values were quite reproducible (RSD ≤ 15%; *n* = 3) considering the measurement set-up and the analyte in the aerosol phase, and a clear exponential trend of the treatment is evidenced.

The treatment severity toward the different Lm strains is pointed out in Fig. 5B, where a strain-dependent behavior is clear; all the Lm strains were significantly affected by the aerosol treatment, in particular, the type strain (Lm ATCC 7644) evidenced a complete inhibition (Log CFU mL^−1^ ˂ 1.99) starting from the lower H_2_O_2_ amount used. On the other hand, the Lm 388 and Lm 641/6II demonstrated a higher resistance, with lethal effects highlighted at treatments conducted at 360 mg L^−1^ and 720 mg L^−1^, respectively. Noteworthy, the Lm 641/6 II pointed out a proportional inhibition at increasing biocide concentrations, with a behavior opposite to the intensity of treatment (Figure S6). EU’s recommendation for biocidal product use [20] suggests H_2_O_2_ 5 min treatments to ensure a final environmental concentration of 360 mg mL^−1^, followed by an “incubation” period of 90 min [23]; higher resistance is usually reported for Lm isolated from nosocomial environment and foods, concerning type strain [23]. The strain-dependent resistance is evident; Lm 641/6II and Lm 338 are characterized by a lower sensitivity to the treatment, as can be inferred by the growth ability, compared to the type strain which is completely inhibited at the EU’s recommended treatment condition.

The ability of the sensors to monitor treatments performed at different times was also assessed, settling the final ambiental concentration at 360 mg mL^−1^; to this aim, nebulization treatments with fogging durations of 2.5, 5, 10, and 15 min were carried out. Figure 5C, reports the Q extrapolated from the amperometric curves (see figure inset). The sensors can monitor H_2_O_2_ fogging performed at different times, returning acceptably reproducible data (RSD ≤ 13%; *n* = 3) up to 15 min. An exponential trend among Q and time of treatment was observed, due to the higher saturation of the environment at increasing treatment times.

Figure 5D reports the inhibition degree obtained for the different Lm strains, subject to treatments carried out at different times. As expected, only slight inhibitions were observed for the shorter treatment for all the strains, while, coherently to previous results and in agreement with the EU recommendations [20], the treatment is effective both for Lm ATCC 7644 and Lm 338 setting the operation time at 5 min. Figure 5E shows the inhibition effect of treatment performed according to the EU recommendation (360 mg L^−1^, 5 min) on the most sensitive (ATCC 7644) and most resistant (641/6II) Lm strains. Noteworthy, Lm 641/6II further demonstrates to possess a stronger resistance, as even the longer time is not able to give effective inhibitions; despite this, for this strain, a decrease in the initial microbial load was observed for increasing treatment times, without reaching total inhibition.

The collected data are coherent with the literature, where different resistances are reported for hydrogen peroxide vapor treatment towards different Lm strains [23]; the two non-type strains possess a greater resistance to H_2_O_2_, compared to the type strain Lm ATCC 7644. In particular, the Lm 641/6II greater resistance can be attributed to adaptive survival mechanisms developed in the environment from which it has been isolated (i.e., a smoked salmon supply chain). Nevertheless, it must be emphasized that the disinfection treatment performed according to the recommendation (5 min nebulization to reach 360 mg L^−1^ H_2_O_2_) is effective for the Lm ATCC 7644 type strain generally used as target microorganisms for disinfection protocols [23].

Summing up, the proposed sensor can differentiate and monitor no-touch disinfection environmental treatments, allowing the modulation of the “treatment intensity” depending on the degree of inhibition required according to the target type strain to inhibit, evidencing treatments not properly carried out; moreover, the sensor can be useful to monitor the treatment close to critical points such as areas of the factory that are difficult to access, or work tools/equipment at high risk of contamination (e.g., blades, punches, working surfaces, etc.). Therefore, by carrying out adequate tests using the target microorganism to inhibit as a model, critical treatment thresholds can be easily settled, and the treatments monitored using the proposed sensor as a point-of-need device.

## Conclusions

For the first time, a 2D/0D heterostructured rGO-nCe conductive film is produced in a single step using a CO_2_ laser-based approach. The laser-assisted synthesis requires a few seconds and the resulting nanostructured film combines the rGO structural and electron-transfer feature with the nanozymatic activity of the nCe. The CO_2_ laser plotter allows to pattern of rGO-nCe films with needed design with micrometric resolution, enabling overcoming cumbersome approaches to synthesize and integrate catalytic nCe in commercial electrodes. The catalytic films were integrated into complete nitrocellulose lab-made sensors brought within everyone’s reach benchtop technologies, resulting in low cost and compatibility with commercial portable potentiostats.

The rGO-nCe sensor allows the direct, sensitive, and reproducible determination of H_2_O_2_ thanks to its catalase-like activity. rGO-nCe sensor was successfully applied for the on-site monitoring of no-touch fogging in-door disinfection treatments toward the effect of hydrogen peroxide on the relevant strain-dependent resistance of Listeria monocytogenes, used as biological targets; for this purpose, Listeria monocytogenes type strain, and strains isolated from food matrix and nosocomial environment were employed.

Impressively, the sensors allow the continuous monitoring of the fogging, proving the ability to distinguish treatments conducted, becoming a useful point-of-need device to differentiate and monitor in-door no-touch disinfection treatments, allowing the modulation of the “treatment intensity” depending on the target bacteria strain to inhibit, highlighting treatments not properly carried out.

### Electronic supplementary material

Below is the link to the electronic supplementary material.

Supplementary Material 1

## Data Availability

Data will be made available on request.
