# Supplementary material for: Laser-induced 2D/0D graphene-nanoceria freestanding paper-based films for on-site hydrogen peroxide monitoring in no-touch disinfection treatments
Source: Mikrochim Acta. 2024 Jun 1;191(6):361. doi: 10.1007/s00604-024-06427-9 (PMC11144143; doi:10.1007/s00604-024-06427-9)
Supplement: Supplementary file 1 — Supplementary Material 1 [file 604_2024_6427_MOESM1_ESM.pdf]

ELECTRONIC SUPPLEMENTARY MATERIAL

Laser-induced 2D/0D graphene-nanoceria freestanding paper-based films for on-site hydrogen peroxide monitoring in no-touch disinfection treatments

José M. Gordón Pidal[a,b,†], Selene Fiori[b,†], Annalisa Scroccarello[b,†], Flavio Della Pelle[b,*], Francesca Maggio[b], Annalisa Serio[b], Giovanni Ferraro[c], Alberto Escarpa[a,**], Dario Compagnone[b,***]

[a]Department of Analytical Chemistry, Physical Chemistry and Chemical Engineering, University of Alcalá, Alcalá de Henares, 28871 Madrid, Spain

[b]Department of Bioscience and Technology for Food, Agriculture and Environment, University of Teramo, Campus "Aurelio Saliceti" Via R. Balzarini 1, 64100, Teramo, Italy

[c]Department of Chemistry "Ugo Schiff" and CSGI, University of Florence, Via Della Lastruccia 3, Sesto Fiorentino, I-50019, Florence, Italy

Corresponding authors contacts:

*fdellapelle@unite.it; **alberto.escarpa@uah.es; *** dcompagnone@unite.it

† J.M.G.P., S.F, and A.S. contributed equally to this paper

TABLE OF CONTENTS

Pg.

SUPPLEMENTARY SECTIONS
SM.2 Material and Methods 3
SM.2.2 Apparatus 3
SM.2.4 Morphochemical and electrochemical characterization 3
SM.2.5 Bacterial strains and cultural conditions

SUPPLEMENTARY SCHEMES
Scheme S1. Flowchart of the rGO-nCe sensors manufacturing. 5
Scheme S2 Graphical sketch of the fogging box and measuring set-up 7

SUPPLEMENTARY FIGURES
Figure S1. Cyclic voltammetry of the full set of sensors in $[Fe(CN)_6]^{3-/4-}$ and $[Ru(NH_3)_6]^{2+/3+}$ 7
Figure S2. Elemental analysis of GO, rGO, and rGO-nCe films. 8
Figure S3. Raman and FTIR of rGO-nCe$_{10}$ film. 9
Figure S4. Cyclic voltammetry of the full set of sensors in H_2O_2 10
Figure S5. Amperometric current obtained for rGO-nCe$_{10}$ sensor in H_2O_2 at increasing working potential 11
Figure S6. Electric charge values extrapolated from fogging treatments performed at different nebulization time 12

SUPPLEMENTARY TABLE
Table S1. Analytical features and figures of merit of sensors present in the literature. 13

SUPPLEMENTARY REFERENCES 14

SUPPORTING SECTIONS

SM.2 Material and Methods

SM.2.2 Apparatus

A Rayjet50 laser machine from Trotec (Wels, Austria) equipped with a CO_2 laser (10.6 µm, 30 Wcw, laser spot of 0.04 mm) was employed to synthesize the rGO-nCe film; the film design was realized with Adobe Illustrator 2020 software. Ink-printing vinyl stencil masks were designed with Silhouette Studio 4.4 basic edition software and patterned using a Cameo 4 craft-cutter machine (Silhouette America®, Lindon, USA). The curing of the inks was performed with a 1700W POL-EKO-APARATURA sp.j. drying oven (ul. Kokoszycka 172C 44 – 300 Wodzisław Śląski). A Specac Atlas Manual 15Ton (15T) hydraulic press (Specac, Orpington, UK) was used for the rGO-nCe film transfer. Electrochemical measurements were performed with a Palmsens 4 potentiostat (Palm Instruments BV, Houten, Netherlands) governed by PSTrace 5.9 software.

SM.2.4 Morphochemical and electrochemical characterization

Morphological and elemental analyses were carried out through scanning electron microscopy (SEM) by using a GeminiSEM 500 (Zeiss Co., Oberkrochen, Germany) equipped with Oxford Aztec Live Microanalysis system with detector Ultim Max 100 (EDS OXFORD, Buckinghamshire, UK) for energy dispersive X-ray spectrometry (EDX) analysis. Micrographs were collected using two different detectors to discriminate elements with high (i.e., Ce) and low (i.e., C) molecular weight; to this aim, an in-Lens detector for secondary electrons and an energy-selective backscatter (ESB) detector for high-angle backscattered electrons in the energy-selected mode were employed. Raman spectra were obtained by a Renishaw inVia™ Qontor® confocal Raman microscope equipped with a Leica DM microscope with a 50LX objective (NA 0.50, WD 8.20 mm). Experiments were performed using the 532 nm laser line (diode-type, Renishaw 80 mW, 532 nm laser, 1800 l mm^{-1} grating) calibrated on the internal Si-reference standard (520.5±0.1 cm^{-1}): spectra were collected with a resolution of 2 cm^{-1} in the spectral range 200-5500 cm^{-1}.

Fourier-transform infrared spectroscopy (FTIR) was carried out with Bruker VERTEX 70v vacuum spectrometer equipped with Transit Platinum ATR (ATR Bruker) with a single reflection diamond crystal and edged in tungsten carbide to purge and seal the beam path of Globar source for the mid-infrared spectral range (MIR) and tungsten-halogen source lamp for the near-IR&Visible spectral range. Opus software and FTIR-ATR Bruker library were employed for the experiments/instruments management, data acquisition, and spectra comparison.

Electrochemical features were explored using Cyclic Voltammetry (CV). CVs were performed using 5 mM $[Fe(CN)_6]^{3-/4-}$ in 0.1 M KCl as inner-sphere redox probe (E range: -0.2 / +0.6 V) and 5 mM $[Ru(NH_3)_6]^{2+/3+}$ in 0.1 M KCl as outer-sphere redox probe (E range: -0.45 / +0.25 V).

SM.2.5 Bacterial strains and cultural conditions

Microbiological disinfection studies were conducted on three *Listeria monocytogenes* (Lm) strains: Lm ATCC 7644 serotype 1/2c type strain (Lm ATCC 7644), Lm 338 serotype 1/2b clinical strain (Lm 338), Lm 641/6II serotype 1/2a food strain isolated from brine injected cold smoked salmon (Lm 641/6II). All strains belong to the collection of the Department of Bioscience and Technology for Food, Agriculture and Environment of Teramo University (Italy), and were stored at -80 °C in TSB added of 20.0% v/v glycerol as cryoprotectant. Before each experiment, the bacterial strains were cultivated overnight at 37 °C in TSA in the Petri dish. Inocula were prepared by taking one colony from the Petri dish, resuspending it in 1 mL of TSB, then incubated at 37 °C for 18h to reach the early stationary phase. Afterward, the cells were harvested by centrifugation and washed three times with 10 mM PBS (pH 7.4).

Then, the inocula were standardized at OD 620nm to reach about 10^8 CFU mL^{-1}, verified through plate count by using the selective medium ALOA at 37 °C. Eventually, 100 µL of the standardized bacterial suspensions were added on a coupon of stainless steel (AISI 304, 2x2x0.1 cm), previously cleaned, and sterilized at 121 °C for 15 min. The inocula on the coupons were let to dry under a biosafety cabinet class II for about 1h. For each strain, coupons in triplicate were prepared.

SUPPLEMENTARY SCHEMES

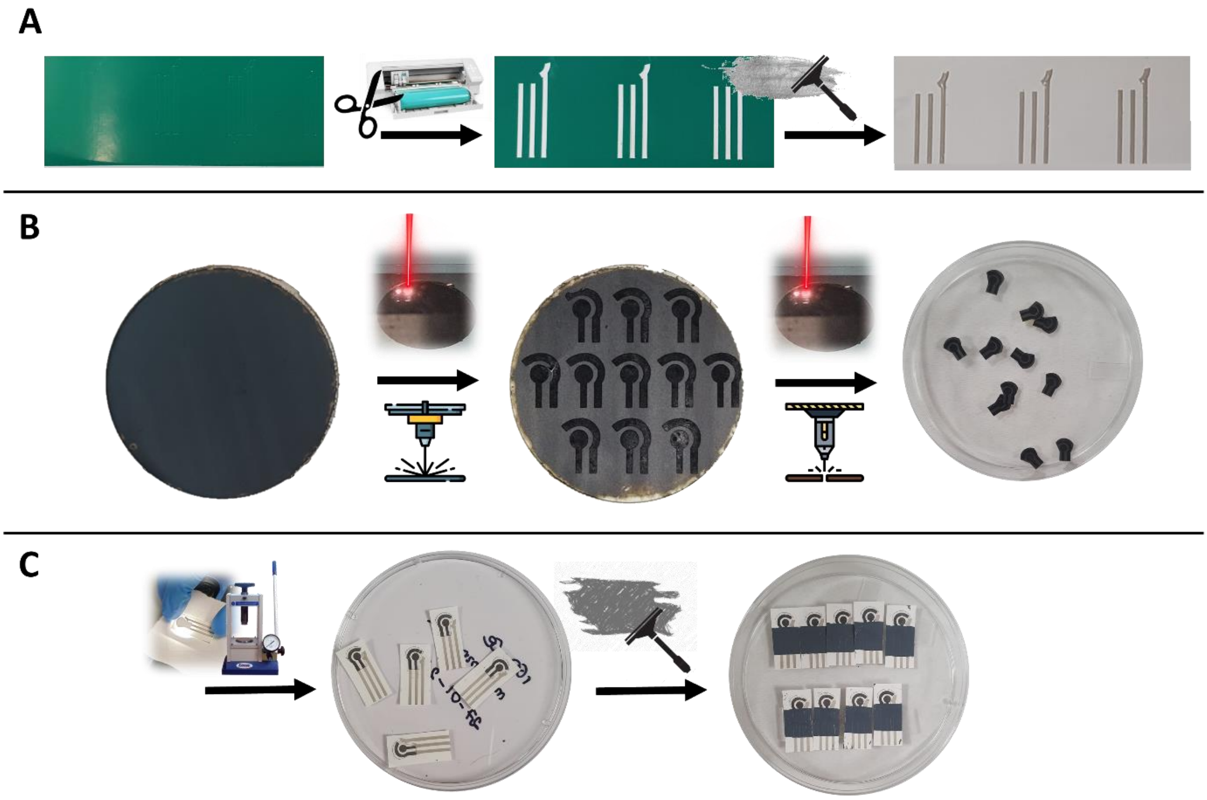

Scheme S1. Main manufacturing steps of the rGO-nCe sensor. (**A**) Vinyl stencil mask manufacturing through a cutter plotter and silver contacts stencil-printing onto the nitrocellulose substrate. (**B**) GO-Ce^{3+} film; laser-induced rGO-nCe formation by engraving; WE and CE cutting by laser in cutting mode. (**C**) rGO-nCe film transferring onto the NTR sensor base and contacts insulation.

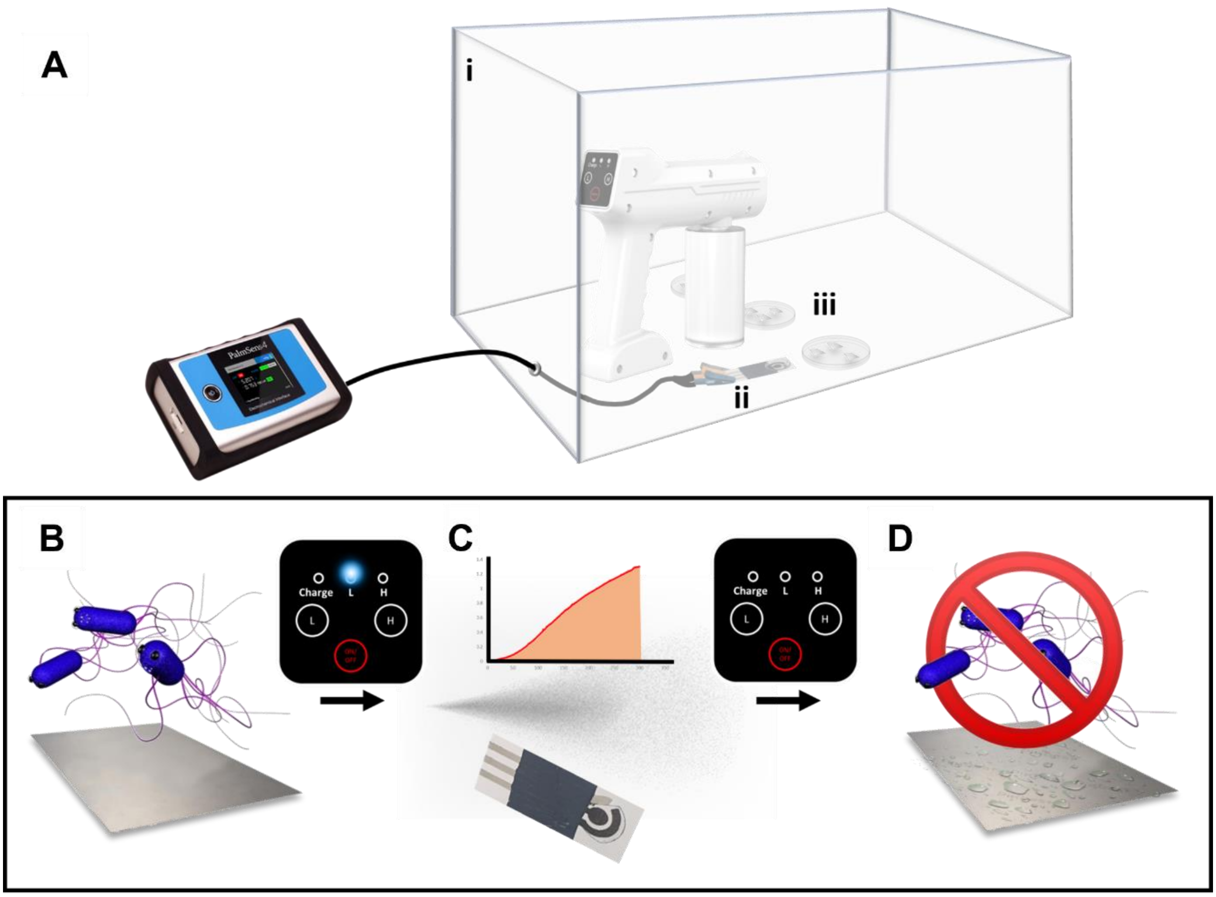

Scheme S2. (**A**) Graphical sketch of the fogging box and measuring set-up. Measurement set-up enclosing the (i) sealed fogging box holding the Portable Nano Atomizer, the (ii) rGo-nCe sensor, and (iii) stainless steel coupons containing the Lm strains. (**B**) coupons containing the vital Lm strains; (**C**) fogging treatment and rGO-nCe sensor-based real-time monitoring via amperometry; (**D**) coupons containing the inhibited Lm strains.

SUPPLEMENTARY FIGURES

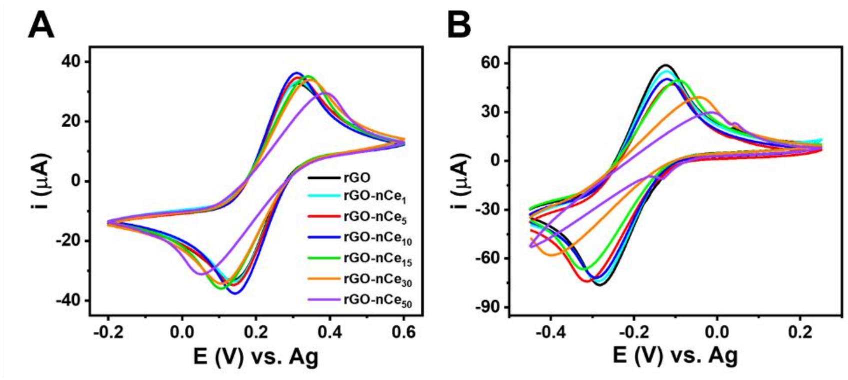

Figure S1. Cyclic voltammograms obtained in (**A**) 5mM $[Fe(CN)_6]^{3-/4-}$ in 0.1 M KCl and (**B**) 5 mM $[Ru(NH_3)_6]^{2+/3+}$ in 0.1 M KCl at 25 mV s^{-1}, using rGO-only sensor and rGO-nCe sensors obtained with different concentrations of Ce^{3+} precursor.

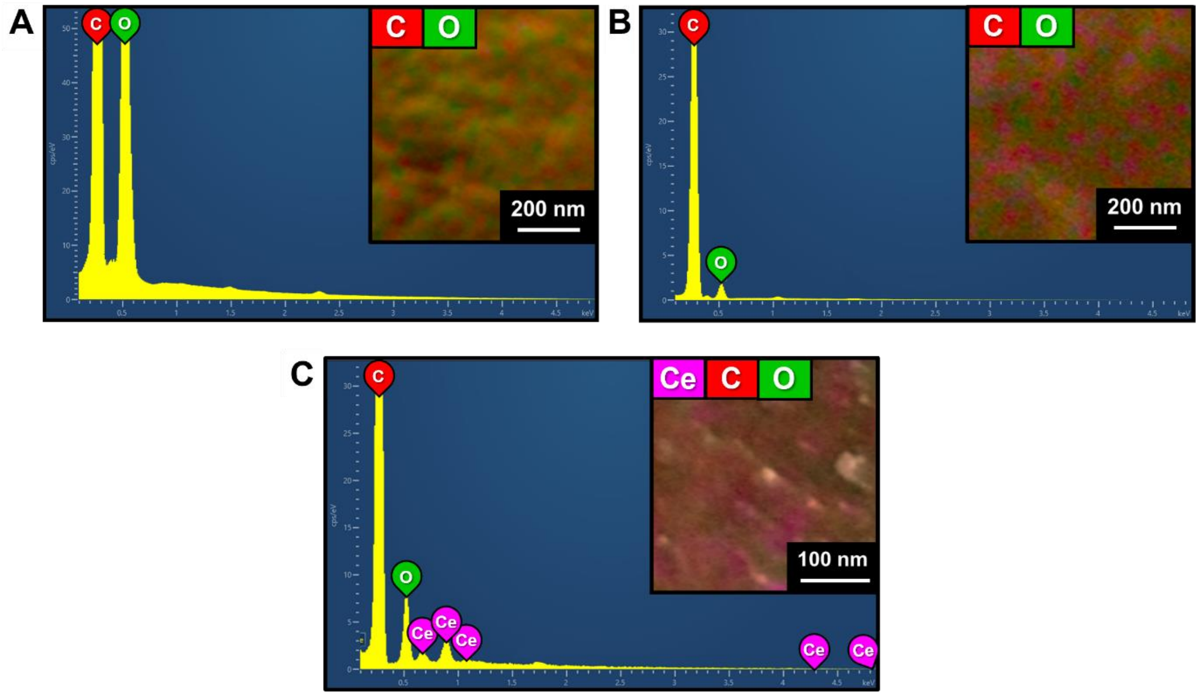

Figure S2. EDX spectra and relative Ce, C, and O elemental mapping for **(A)** GO, **(B)** rGO, and **(C)** rGO-nCe_{10} films.

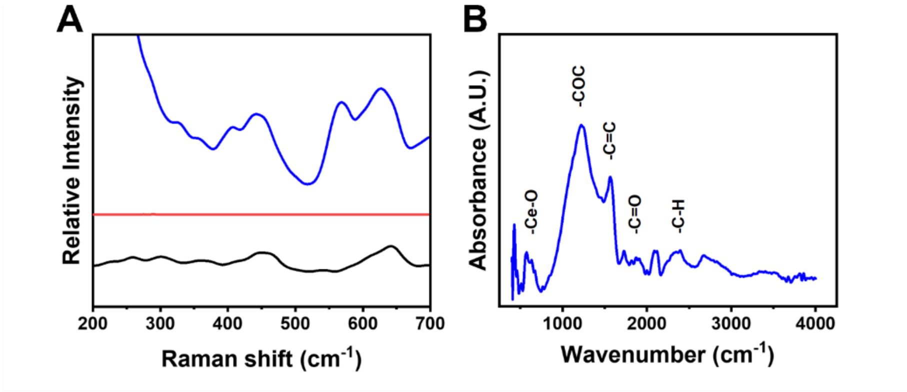

Figure S3. (A) Raman spectra of GO (black line), rGO (red line), and rGO-nCe10 (blue line) films obtained between 200-700 cm^{-1}. (**D**) FTIR spectrum of the rGO-nCe_{10} acquired in the transmission mode.

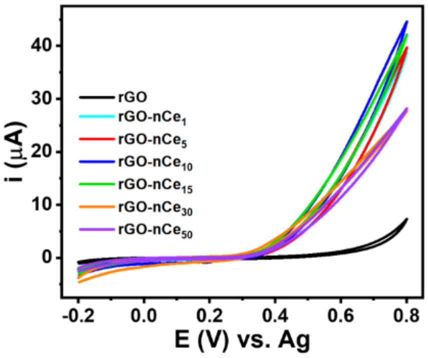

Figure S4. Cyclic voltammograms obtained in 10 mM H_2O_2 in PB at 25 mV s^{-1} for rGO and rGO-nCe sensors obtained with different concentrations of Ce^{3+} precursor.

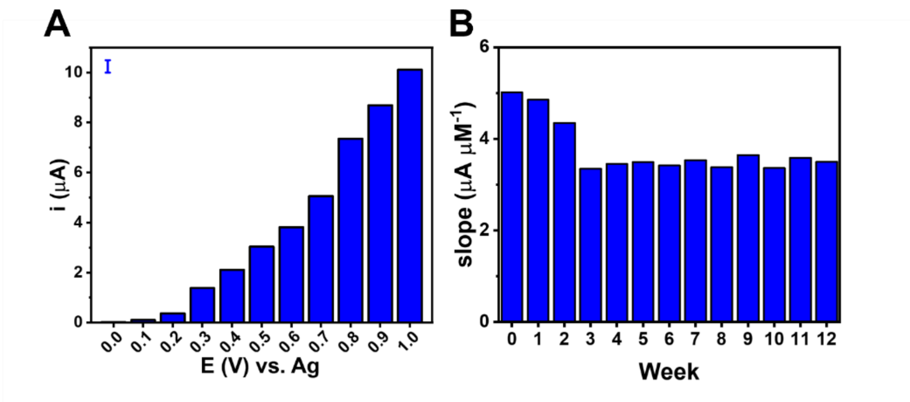

Figure S5. (**A**) Amperometric currents were obtained at increasing potentials with the rGO-nCe$_{10}$ sensor in 400 μM H_2O_2 in PB. (**B**) Linear regression slope of calibration curves obtained by using rGO-nCe$_{10}$ sensors at different storage times.

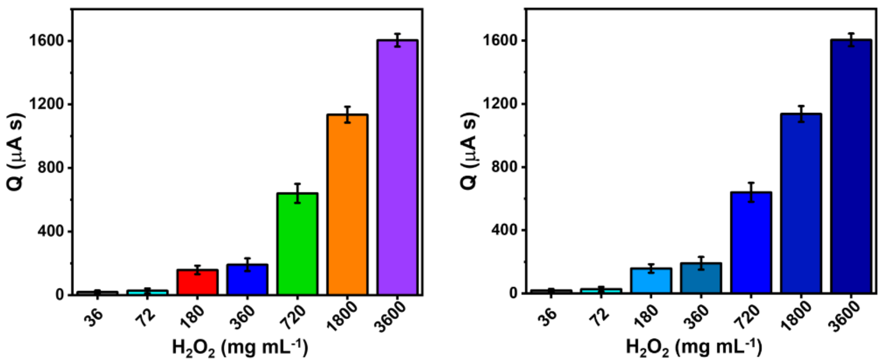

Figure S6. Electric charge values (Q) extrapolated from 5 min fogging treatments, were performed to ensure increasing environmental concentrations of the nebulized H_2O_2. The amperometric curves from which the data are extrapolated are reported in Figure 5A.

SUPPLEMENTARY TABLE

Table S1. Analytical features and figures of merit of sensors/electrode enclosing nCe for H_2O_2 determination

Transducer	Sensing material	Sensor manufacturing	Method	Work. potential (V)	L.R. (μM)	LOD (μM)	Application	Ref.
ITO	Peroxidase immobilized onto NanoCeO_2	1) Commercial CeO_2 dispersion in EtOH 2) CeO_2 film deposition 3) HRP immobilization	Cyclic voltammetry	-	1-170	0.5	-	[1]
GCE	Polycrystalline Cerium Oxide	1) Commercial CeO_2 film formation via magetron sputtering onto GCE	Amperometry	0.65	5-460	5.0	-	[2]
3DG/NF	CeNP - graphene	1) Graphene synthesis via CVD 2) Commercial CeNP drop-casting	Cyclic voltammetry	-	2.8-160	1.0	-r	[3]
GCE	Hb/CeO_2/MWNTs/CHIT	1) CeO_2 dispersion in MWNTs/CHIT solution 2) CeO_2/MWNTs/CHIT drop-casting onto GCE 3) Hb deposition	Cyclic voltammetry	-	5-460	0.7	-	[4]
GCE	Mb/CeO_2@MWNTs	1) CeO_2@MWNTs nanocomposite ultrasonication and thermal synthesis 2) Mb and CeO_2@MWNTs solutions mixing 3) Drop-casting onto GCE	Amperometry	-0.34	1-143	0.2	-	[5]
SPE	GS@CeO_2-Pt	1) GS@CeO_2 nanocomposites synthesis via solvothermal process 2) GS@CeO_2 drop-casting onto SPE 3) SPE immersion in ethylenediamine and H2PtCl_6·6H_2O solution and Pt reduction	Amperometry	-0.3	1-10000	0.4	H_2O_2 in contact lens care solutions	[6]
GCE	Polyaniline/cerium oxide	1) CeO_2 hydrothermal synthesis 2) Oxidative polymerization of pure PANI 3) In situ polymerization of PANI/CeO_2 nanocomposite 4) PANI/CeO_2 deposition onto GCE	Linear sweep voltammetry	-	2-100	0.2	H_2O_2 in milk and tap water	[7]
Paper-based sensor integrating a rGO-nCe film	rGO-nCe film	1) rGO-nCe film formation via CO_2 laser 2) rGO-nCe integration into lab-made nitrocellulose sensors	Amperometry	0.4	0.5-1500	0.3	No-touch disinfection continuous monitoring	**This work**

ITO= indium-tin-oxide; HRP= horseradish peroxidase; GCE= glassy carbon electrode; 3DG/NF= Three-dimensional graphene/ nickel foam; Hb/CeO_2/MWNTs/CHIT= hemoglobin/CeO_2/multi-walled nanotubes/chitosan; SPE= screen printed electrode; GS@CeO_2-Pt= graphene sheets@cerium oxide-platinum; PANI= Polyaniline; PBS= phosphate buffer saline

SUPPLEMENTARY REFERENCES

[1] A. A. Ansari, P. R. Solanki, and B. D. Malhotra, “Hydrogen peroxide sensor based on horseradish peroxidase immobilized nanostructured cerium oxide film,” *J. Biotechnol.*, vol. 142, pp. 179–184, 2009, doi: 10.1016/j.jbiotec.2009.04.005.

[2] Y. Kosto *et al.*, “Electrochemical activity of the polycrystalline cerium oxide fi lms for hydrogen peroxide detection,” *Appl. Surf. Sci.*, vol. 488, no. May, pp. 351–359, 2019, doi: 10.1016/j.apsusc.2019.05.205.

[3] C. N. Macambira *et al.*, “Synthesis , First-Principle Simulation , and Application of Three-Dimensional Ceria Nanoparticles / Graphene Nanocomposite for Non- Enzymatic Hydrogen Peroxide Detection,” *J. Electrochem. Soc.*, vol. 166, no. 5, pp. 3167–3174, 2019, doi: 10.1149/2.0191905jes.

[4] X. Zhang, B. Qi, and S. Zhang, “Direct Electrochemistry of Hemoglobin in Cerium Dioxide / Carbon Nanotubes / Chitosan for Amperometric Detection of Hydrogen Peroxide,” *Anal. Lett.*, vol. 41, no. 17, pp. 37–41, 2008, doi: 10.1080/00032710802463055.

[5] J. Qiu, S. Cui, and R. Liang, “Hydrogen peroxide biosensor based on the direct electrochemistry of myoglobin immobilized on ceria nanoparticles coated with multiwalled carbon nanotubes by a hydrothermal synthetic method,” *Microchim. Acta*, vol. 171, pp. 333–339, 2010, doi: 10.1007/s00604-010-0440-z.

[6] X. Yang, Y. Ouyang, F. Wu, Y. Hu, H. Zhang, and Z. Wu, “In situ & controlled preparation of platinum nanoparticles dopping into graphene sheets @ cerium oxide nanocomposites sensitized screen printed electrode for nonenzymatic electrochemical sensing of hydrogen peroxide,” *JEAC*, vol. 777, pp. 85–91, 2016, doi: 10.1016/j.jelechem.2016.08.008.

[7] M. A. Hussein, A. Khan, and K. A. Alamry, “A highly efficient electrochemical sensor containing polyaniline/cerium oxide nanocomposites for hydrogen peroxide detection,” *RSC Adv.*, vol. 12, no. 49, pp. 31506–31517, 2022, doi: 10.1039/d2ra05041b.
